# Hemodynamic Characteristics of Patients With Suspected Coronary Heart Disease at Their Initial Visit

**DOI:** 10.3389/fphys.2021.714438

**Published:** 2021-07-20

**Authors:** Haoyao Cao, Yiming Li, Yiming Zhao, Tianyuan Xiong, Zhan Liu, Tinghui Zheng, Mao Chen

**Affiliations:** ^1^Department of Applied Mechanics, Sichuan University, Chengdu, China; ^2^Department of Cardiology, West China Hospital, Sichuan University, Chengdu, China

**Keywords:** coronary artery stenosis, hemodynamics, coronary computed tomographic angiography, Initial diagnosis, clinical outcome

## Abstract

**Purpose:**

It is difficult for doctors to decide whether patients with suspected coronary heart disease classified as Coronary Artery Disease Reporting and Data System (CAD-RADS) < 3 should be administered preventive treatment, or whether non-atherosclerotic chest pain should be considered. The aim of the current study was to investigate coronary hemodynamic characteristics in such patients, which may provide more information on their stenosis and be helpful for initial diagnoses.

**Methods:**

Two patient-specific models were reconstructed based on the coronary computed tomographic angiography underwent in 2012. Patient 1 was classified as CAD-RADS 0, and was readmitted to hospital due to coronary artery disease within 5 years. Patient 2 was classified as CAD-RADS 2, and has experienced no adverse events to date. Computational fluid dynamics (CFD) analysis was used to obtain hemodynamic parameters including flow rate waveform, flow streamlines, time-average wall shear stress (TAWSS), and oscillatory shear index (OSI).

**Results:**

Patient 1 exhibited no physiological characteristics of right coronary artery flow waveform, large areas of low TAWSS, and slow blood flow in the proximal and middle segments of the left anterior descending branch. Patient 2 exhibited reduced coronary supply, small and separate areas of abnormal TAWSS, and a higher left anterior descending branch OSI than patient 1.

**Conclusion:**

Hemodynamic abnormalities may play an important role in the prognosis of patients with coronary stenosis, and patient-specific hemodynamic characteristics may facilitate more accurate initial diagnosis, and better management. Overall hemodynamics (along the whole vessel) warranted attention at the time of the initial visit in patients classified as CAD-RADS < 3.

## Introduction

Coronary artery stenosis is one of the most contributory components of acute coronary syndrome and sudden cardiac death. Coronary computed tomographic angiography (CCTA) is usually used to observe coronary lumen shape and plaque characteristics in patients with suspected coronary heart disease at the time of their initial visit (Helfant et al., 1970; Motoyama et al., 2009; Saremi and Achenbach, 2015). Coronary stenosis degree is currently usually assessed based on lumen diameter reduction. The Coronary Artery Disease Reporting and Data System (CAD-RADS)—based on the highest-grade stenosis recorded by CCTA—provides specific suggestions for further management of patients with suspected coronary heart disease (Cury et al., 2016). Patients classified as CAD-RADS ≥ 3 (stenosis degree ≥ 50%) are diagnosed with “coronary heart disease” and administered a widely accepted planning treatment aimed at avoiding the occurrence of myocardial ischemia, hypoxia, and necrosis (Miller et al., 2008; Alexopoulos et al., 2010; Roffi et al., 2015; Cury et al., 2016). Controversy arises in patients classified as CAD-RADS < 3 (stenosis degree < 50%), however, because both preventive therapy and or non-atherosclerotic causes of chest pain need to be considered (Cury et al., 2016). In most patients classified as CAD-RADS < 3 their condition is effectively controlled after the administration of timely drug treatment. Notably, however, there are some patients in whom the risk of coronary heart disease is excluded based on “minimal” coronary stenosis at the time of their initial diagnosis, but who nonetheless go on to suffer coronary artery plaque after a period of months or years without anti-atherosclerosis treatment (Pflederer et al., 2010; Lee et al., 2019). Therefore, accurate diagnosis and timely treatment of patients classified as CAD-RADS < 3 is crucial.

Hemodynamic evaluation is useful for coronary artery disease. Fractional flow reserve is currently widely used to assess the functional severity of moderate coronary stenosis (CAD-RADS ≥ 3), with the aim of confirming the influence of the stenosis on the myocardial ischemia (Hulten et al., 2013; Lee et al., 2018). In patients classified as CAD-RADS < 3, however, it may be that plaque growth and thrombosis warrant more attention than coronary supply.

In recent years there has been growing interest in the use of computational fluid dynamics (CFD) analysis based on patient-specific CCTAs in the field of cardiovascular disease (Taylor et al., 2013; Zarins et al., 2013). Compared to traditional clinical imaging, CFD analysis provides quantitative hemodynamic data linked to thrombotic and atherosclerotic risk, including intravascular and near wall flow features such as the streamlines of blood flow, time-averaged wall shear stress (TAWSS), and oscillatory shear index (OSI). It has also been used extensively in clinical applications such as abdominal aortic aneurysm rupture risk prediction (Qiu et al., 2018), investigation of the hemodynamic effects of morphologic stenosis parameters on renal artery stenosis (Xiong et al., 2021), and the choice of occlusion position in cases of coronary artery fistula (Cao et al., 2020).

Previous patient-specific hemodynamic evaluations of severe coronary stenosis based on CCTA focused on myocardial ischemia and plaque rupture (Johnson et al., 2014; Kang et al., 2018; Park et al., 2019). Whether the hemodynamic characteristics of patients with chest pain suspected of coronary heart disease at the time of their initial visit can provide useful information for their diagnosis, and even the prediction of clinical outcomes, have not been investigated. The current study investigated two representative patients classified as CAD-RADS < 3. Patient-specific CFD based on their initial-visit CCTA were analyzed in an effort to identify the key hemodynamic information, with the ultimate aim of providing some theoretical support for diagnoses and treatments.

## Materials and Methods

### Study Population

In the current study two hospitalized patients who underwent CCTA examination at a single center in 2012 were retrospectively analyzed. Patient-specific clinical records were provided by the West China Hospital of Sichuan University (Chengdu, Sichuan, China) and included computed tomography image data. Assessment of high-risk signs and quantitative analysis were performed by two independent cardiac radiologists. The study was conducted in accordance with the principles of the Declaration of Helsinki and met the relevant medical ethics requirements. The Ethical Review Committee of the West China Hospital of Sichuan University approved the study.

Patient 1, a 77-year-old female, presented at the hospital due to chest pain. Considering the history of hypertension and the family history of coronary artery disease, the coronary artery-related examinations were performed in this patient. CCTA did not indicate any substantial stenosis in the three main coronary arteries. Discharge medications included anti-hypertension and nitrate drugs. Subsequent follow-up via a telephone interview revealed that he was readmitted to a local hospital due to coronary artery disease 5 years after discharge.

Patient 2 was an 80-year-old male who underwent CCTA prior to cataract surgery. Mild to moderate stenosis of the left anterior descending branch was detected. Discharge medication included the statin, the beta-blocker, and the aspirin. To follow-up date (September 2017) the patient has not experienced any adverse events. Basic information about the two patients at the time of their initial visits is shown in Table 1.

**TABLE 1:** Basic information of patients at the time of initial visit.

	Patient 1	Patient 2
Blood pressure (mmHg)	140/66	165/78
CAD-RADS	0	2
Location of stenosis	–	LAD, RCA
Max degree of stenosis (%)	–	29.32
Min lumen diameter at stenosis (mm)	–	1.66
Clinical outcome	Readmission of coronary heart disease	None

**FIGURE 1:**
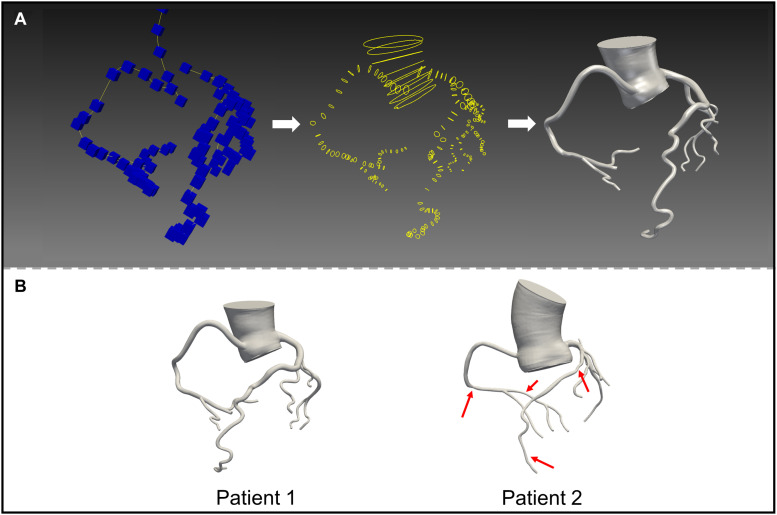
3D reconstructed mode ls based on computed tomography images. **(A)** Construct the centerlines of the vessels (left); segment the vessel perpendicular to the centerline (middle); loft the segmentations (right). **(B)** The models of patient 1 and 2.

Thin-slice computed tomography angiography (CTA) images of the two patients were obtained using a second-generation dual source computed tomography system (SOMATOM Definition CT, Siemens Medical Solutions; Forchheim, Germany). The slice thickness of the images was 0.6 mm. Patient-specific three-dimensional anatomical models including the coronary tree and the aortic root were reconstructed from CTA data using an open-source SimVascular software package (Figure 1; Updegrove et al., 2017). In accordance with the SimVascular tutorial and the mesh independency study, the sizes of the finite volume mesh were set to 0.05 mm for the coronary arteries and 0.2 mm for the aorta. Four boundary layers were used with 0.6 as the height ratio and 0.05 mm as the initial height. Patient 1 had resulting meshes of 1,176,121 unstructured triangular elements, and patient 2 had resulting meshes of 1,237,962 unstructured triangular elements. Rigid vessel walls were assumed.

### CFD Boundary Conditions

**FIGURE 2:**
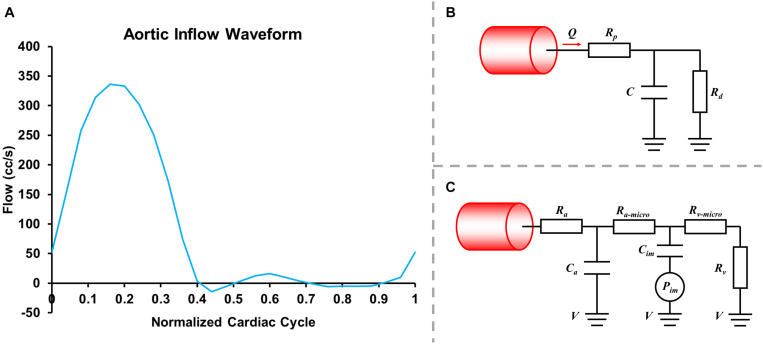
Inlet flowrate wave **(A)**, Windkessel RCR boundary conditions **(B)** and lumped parameter network (LPN) coronary model **(C)**.

In the current study blood was assumed to be incompressible, laminar, unsteady, homogenous, and Newtonian. The corresponding governing equations were:

(1) $$\frac{\partial \vec{\text{u}}}{\partial \text{t}}+\rho \left(\vec{\text{u}}\cdot \nabla \right)\vec{\text{u}}+\nabla \text{p}-\mu \Delta \vec{\text{u}}=0$$

(2) $$\nabla \cdot \vec{\text{u}}=0$$

where $\vec{u}$, *p*, ρ, and μ, respectively, represent fluid velocity vector, pressure, density (1.06 g/mL), and the dynamic viscosity of blood (0.04 dyne.s/cm^2^). All hemodynamic parameters were analyzed using SimVascular (Updegrove et al., 2017). A normal human flow waveform (Figure 2A), the Windkessel RCR boundary conditions (Figure 2B), and the lumped parameter network coronary model (Figure 2C) were used at the aortic inlet, the aortic outlet and coronary artery outlets, respectively (Kim et al., 2009, 2010; Sankaran et al., 2012). The formulas of boundary conditions were:

(3) $${\text{R}}_{\text{d}}+{\text{R}}_{\text{p}}=\frac{{\text{P}}_{\text{mean}}}{{\text{Q}}_{\text{aorta}}}$$

(4) $${\text{R}}_{\text{cor}}:{\text{R}}_{\text{aorta}}={\text{Q}}_{\text{aorta}}:{\text{Q}}_{\text{cor}}$$

where *R_p_* was the viscous resistance of the downstream arterial vasculature, *R_d_* was the resistance of the capillaries and venous circulation, *P*_mean_ was the mean pressure, *R*_cor_ and *Q*_cor_ represented the total resistance and the cardiac output of coronary arteries (mL/s) and *R*_aorta_ and *Q*_aorta_ represented the total resistance and the cardiac output of the aorta. The resistances for each coronary outlet could be split into *R_a_* (arterial resistance), *R*_a–micro_ (microcirculation resistance), *R_v_* (venous resistance), *C_a_* (microcirculation compliance), *C*_im_ (myocardial compliance), and *P*_im_ (intra-myocardial pressure). The parameters were adjusted based on reference values, coronary morphology, coronary flowrate, and blood pressure. In the current study *R*_*d*_:*R*_*p*_ = 0.91:0.09, and *C*_*a*_:*C*_im_ = 0.11:0.89. The compliance of blood vessels (the capacitor *C*) was 0.001 cm^5^/dyne.

Based on past experience twelve cardiac cycles were set for the simulation, and each pulse cycle was divided into 500 time-steps. The simulations were run until the pressure fields at the inlet and outlet did not change more than 1% from the previous cycle, and the data from the last cycle were selected as the result. Additional details are described in Sengupta et al. (2014) and Cao et al. (2020).

### Hemodynamic Variables

TAWSS can be used to evaluate the wall shear stress acting on the lumen wall under pulsating flow in heart circulation. Abnormal TAWSS (<4 dyne/cm^2^ or > 40 dyne/cm^2^) may cause blood cell aggregation, platelet activation, and inflammatory cell-mediated destructive remodeling (Xiang et al., 2014). TAWSS is defined as:

(5) $$\text{TAWSS}=\frac{1}{\text{T}}\int_{0}^{\text{T}}\left|\text{WSS}\right|\text{dt}$$

where, T is the period of the cardiac cycle, and WSS is the WSS vector.

OSI is a frequently used index to evaluate axial directional change in WSS within the cardiac cycle. Abnormal OSI indicates that the flow field is highly disturbed, which is associated with the formation of thrombosis (Xiang et al., 2014). OSI is defined as follows:

(6) $$\text{OSI}=0.5\times \left[1-\frac{\left|\int_{0}^{\text{T}}\text{WSSdt}\right|}{\int_{0}^{\text{T}}\left|\text{WSS}\right|\text{dt}}\right]$$

where T is the period of the cardiac cycle, and WSS is the WSS vector.

Abnormal exposure [A _(Patient – Position – parameter)%_] was used to show areas of abnormal hemodynamic parameters clearly. For example, A _(1 – LAD – TAWSS < 4)%_ is the ratio of the area that satisfied TAWSS < 4 dyne/cm^2^ to the left anterior descending branch (LAD) area of patient 1.

## Results

### Flow Waveform

**FIGURE 3:**
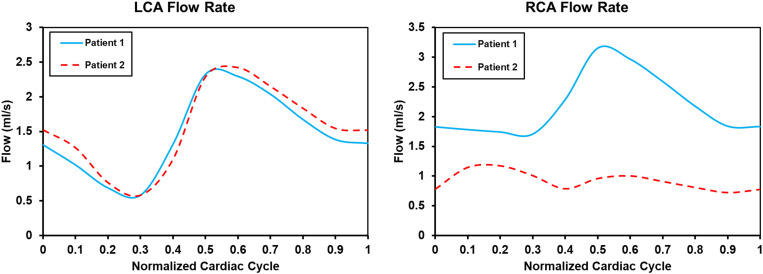
Coronary flow rate waveform in two models.

Flow rate waveforms of the left coronary artery (LCA) and right coronary artery (RCA) in the two patients are shown in Figure 3. In both patients the LCA flow rate waveforms were low in systolic phase and high in diastolic phase. In patient 2 the RCA flow rate waveforms had two characteristic peaks. Thus, the adoption of the boundary conditions successfully captured the physiologic behavior of coronary flow. Intramyocardial pressure in the systole is high to impede blood flow through the coronary arteries, whereas intramyocardial pressure in the diastole is low to facilitate higher flow (Sengupta et al., 2012). The flow rate waveform of the RCA in patient 1 only had one peak. The flow flux to the LCA and RCA, respectively, were 15.96 mL/cycle and 23.89 mL/cycle in patient 1, and 16.98 mL/cycle and 10.05 mL/cycle in patient 2. In patient 1 blood through the coronary artery accounted for 4.12% of cardiac output, and in patient 2 it accounted for 2.80%. The normal flow rate of coronary arteries is approximately 4% of cardiac output. The maximum and minimum LCA flow rates were 2.32 and 0.58 mL/s in patient 1, and 2.42 and 0.58 mL/s in patient 2. The maximum and minimum RCA flow rates were 3.15 and 1.70 mL/s in patient 1, and 1.17 and 0.72 mL/s in patient 2.

### Flow Pattern

**FIGURE 4:**
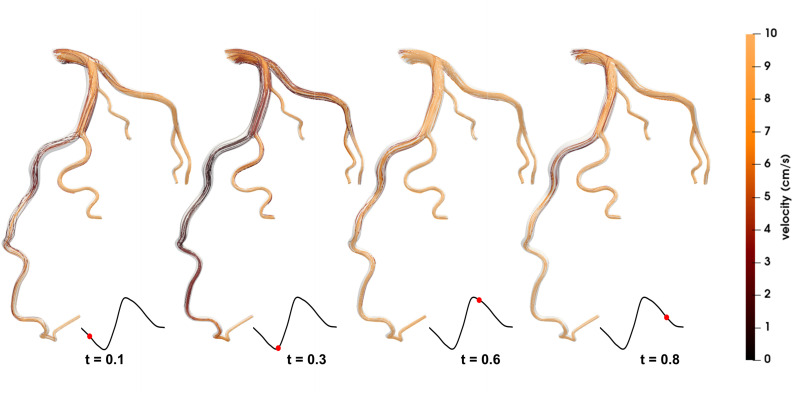
Streamlines of the blood flow of patient 1 are disordered. The red dots on the left coronary flow waveforms indicate the acquisition time of the corresponding images above.

**FIGURE 5:**
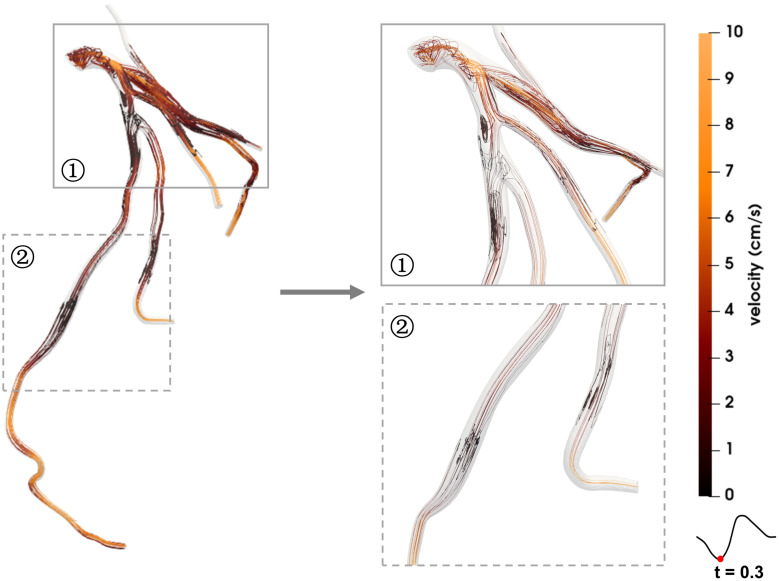
Streamlines of the blood flow disorder of patient 2. The red dots on the left coronary flow waveforms indicate the acquisition time of the corresponding images above.

Figures 4, 5 show the respective streamlines of LCA in patients 1 and 2. In patient 1 blood flow was characterized by a smooth flow channel. There was no obvious blood flow disorder, with low velocity in the proximal and middle LAD during the entire cardiac cycle (Figure 4). The low-velocity flow was more pronounced during the systole (*t* = 0.1, *t* = 0.3) and end diastole (*t* = 0.8). Although the velocity in the center was apparently normal in the diastole (*t* = 0.6), it was quite low and even approached stagnation near the vessel wall. In patient 2, at the systole peak (*t* = 0.3) the flow was helical from the LCA entrance. A small low-speed vortex was evident at the proximal LAD near a branch. Then, at the position before the first stenosis there was a blood flow disturbance with low velocity. At the position between two stenosis lesions the flow velocity was slightly lower than that in other locations. Lastly, at the proximal part of the second stenosis the flow was chaotic and the velocity gradually decreased to almost zero. Compared with patient 1, there were chaotic streamlines evident at the distal parts of other branches except the LAD in patient 2.

### Hemodynamic Characteristics

**FIGURE 6:**
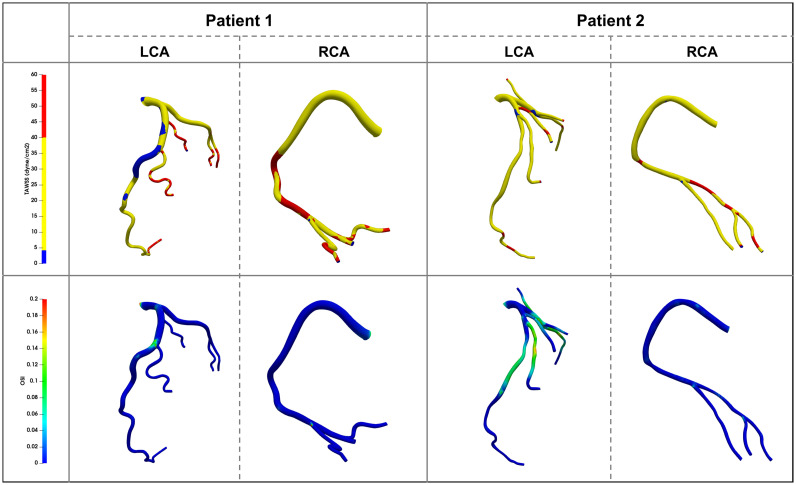
Time average wall shear stress (TAWSS) and Oscillatory Shear Index (OSI) distribution contour map on the vessel walls in two patients.

Figure 6 shows the TAWSS and OSI distributions in the coronary arteries of the two patients. In patient 1 the TAWSS distribution was quite non-uniform. Regions of extremely low TAWSS values (<4 dyne/cm^2^) were evident at the proximal and middle parts of the LAD [A _(1 – LAD – TAWSS < 4)%_ = 24.30%], which was consistent with the low blood flow velocity area shown in Figure 4. Multiple high TAWSS values of up to 40 dyne/cm^2^ were evident at the middle and distal parts of the RCA [A _(1 – *RCA* – TAWSS > 40)%_ = 26.13%], and the maximum value was 147.87 dyne/cm^2^. In patient 2 the most prominent characteristic was small areas of abnormal TAWSS scattering on the vessel walls [A _(2 – LCA – TAWSS < 4)%_ = 5.29% and A _(2 – LCA – TAWSS > 40)%_ = 6.11%, A _(2 – *RCA* – TAWSS > 40)%_ = 12.19%]. In both patients 1 and 2 the ratio of the area of OSI > 0.2 accounted for less than 1%. An increase in OSI was evident in the middle of the LAD in patient 1, and between the two stenoses in patient 2, indicating frequent directional changes over the cardiac cycle. The positions near the branches or after the stenoses were more prone to higher OSIs (Figure 6).

## Discussion

It is difficult to evaluate whether adverse events will occur in patients classified as CAD-RADS < 3 based on the information provided by CCTA at the time of their initial visit. The current study included two representative patients with suspected coronary heart disease. Patient 1 did not take anti-atherosclerosis drugs, and suffered adverse events during follow-up. Patient 2 had preventive atherosclerosis treatment and experienced no adverse events. CFD analysis was used to explore the hemodynamic characteristics of the patients, and may provide useful information for diagnosis and treatment.

The results revealed that both patients 1 and 2 had hemodynamic abnormalities. Specifically, patient 1 exhibited no physiological characteristics of RCA flow waveform. One quarter of the vessel wall areas of the LAD and the RCA exhibited low and high TAWSS, respectively. Large areas of low TAWSS (<4 dyne/cm^2^) and slow blood flow (close to stagnation) appeared at the proximal and middle segments of the LAD. The areas of TAWSS > 40 dyne/cm^2^ were among the middle and distal RCA vessel wall. Patient 2 exhibited reduced coronary supply. The areas of abnormal TAWSS values were small and separate. Patient 2′s OSI was higher than that of patient 1. Previous studies have shown that TAWSS > 40 dyne/cm^2^ or high OSI (no exact value but generally > 0.2 or 0.3) lead to vascular wall damage, whereas TAWSS < 4 dyne/cm^2^ and blood flow disorder can induce extensive platelet aggregation, damage endothelial cells, and lead to thrombosis and atherosclerosis (Kroll et al., 1996; Park et al., 2016; Jahromi et al., 2019). Accordingly, there may be a risk of thrombotic development and plaque growth in the LAD of patient 1. The other left coronary branches and the RCA in patient 1 may have developed further vascular wall damage due to increased flow velocity, which resulted in subsequent readmission for coronary heart disease. Similarly, plaque growth and new plaques appearing between the two stenoses may represent a risk that patient 2 has to face in the future. Based on their hemodynamic characteristics both patients should have been advised to take statin and aspirin at the time of their initial visits, to avoid adverse events.

Previous studies have mainly focused on local hemodynamics abnormalities along the stenoses (Lee et al., 2017; Malota et al., 2018; Wong et al., 2020). The current study suggests that overall hemodynamics (along the whole vessel) are also worthy of attention, however, especially in patients classified as CAD-RADS < 3 at the time of initial diagnosis. In general, the hemodynamic environment interacts with stenosis. For example, in patient 1 nearly 25% of LAD were exposed to low TAWSS, and one quarter of the RCA vessel walls were exposed to high TAWSS. If there was no timely treatment, there would be reason to believe that this abnormal hemodynamic environment would lead to plaque formation and vascular wall damage. The patient was readmitted due to coronary heart disease during follow-up. In patient 2 the flow disturbance and high OSI area were concentrated in the proximal end of the first stenosis and between the two stenoses. In patients classified as CAD-RADS < 3 the overall hemodynamics may be more consequential than local lesions. If only the smallest lumen was noticed, this abnormality would be missed. Therefore, both local and overall hemodynamics need to be investigated during the initial diagnosis, and may have substantial effects on clinical outcomes.

In clinical application, whether hypertension in patients with coronary stenosis should be controlled remains controversial. Some clinical experts have suggested that high blood pressure imposes an extra load on the heart and should be controlled to a normal value (Rosendorff et al., 2007, 2015). Others have argued that it should be cautiously controlled because the survival rate of hypertensive patients was higher than that of normotensive patients (Rosendorff et al., 2007, 2015). CFD analysis indicated that patient 2 would suffer from insufficient coronary supply, but no cardiovascular events were recorded in that patient. Due to the absence of boundary conditions such as heart rate, true aortic inlet flow waveform, and so on, we speculated that hypertension may guarantee perfusion in the case of coronary stenosis. With regard to the use of a beta-blocker, additional follow-up of patient 2 is required, as are future studies with more patients.

Although the sample size was small in the present study, observations such as hemodynamics along the whole vessel are of reference value with respect to patients classified as CAD-RADS < 3 at the time of their initial visit. Based on this preliminary study, our future research will focus on the following: (1) Does the degree of coronary stenosis have a strong effect on coronary blood supply and less of an effect on the blood flow disorder? (2) Does the location of stenosis have a strong effect on intraluminal blood flow disorder? (3) Can similar vascular bed morphologies lead to different clinical outcomes due to different hemodynamic characteristics? (4) More patient-specific measurements, such as patient-specific inlet flow rate waveform, should be used to obtain more convincing results.

## Conclusion

In the current study numerical simulation of two patient-specific coronary models was conducted via CFD methods. Surprisingly the patient classified as CAD-RADS 0 had abnormal hemodynamic characteristics that could not be ascertained from the CCTA directly. The results suggested that this patient should also have been treated effectively at the time of their initial visit, to avoid future adverse events. It is difficult to accurately diagnose a patient and decide on their treatment based solely on highest-grade stenosis recorded via CCTA. Patient-specific hemodynamic characteristics may play as important a role as changes in coronary artery morphology in the prognosis of patients with coronary stenosis. In conclusion, patient-specific CFD analysis may assist initial diagnosis and management, especially of patients classified as CAD-RADS < 3.

## Data Availability Statement

The original contributions presented in the study areincluded in the article/supplementary material. Further inquiries can be directed to the corresponding authors.

## Author Contributions

HC: modeling, simulation, formal analysis, data curation, visualization, writing—original draft. YL: resources, modeling, formal analysis, writing—original draft, funding acquisition. YZ: modeling, data curation, visualization. TX: resources, validation, formal analysis. ZL: methodology, supervision. TZ: conceptualization, methodology, writing—editing, project administration, funding acquisition. MC: resources, validation, writing—editing, project administration, funding acquisition. All authors gave final approval for publication and agree to be held accountable for the work performed therein.

## Conflict of Interest

The authors declare that the research was conducted in the absence of any commercial or financial relationships that could be construed as a potential conflict of interest.
